# Influences of cushion contour on passenger comfort and interface pressure in high-speed train

**DOI:** 10.1371/journal.pone.0276900

**Published:** 2023-02-13

**Authors:** Juan Li, Jifeng Lian, Jiujiang Wu

**Affiliations:** 1 School of Art and Design, Xihua University, Chengdu, Sichuan, China; 2 School of Emergency Management, Xihua University, Chengdu, Sichuan, China; 3 Shock and Vibration of Engineering Materials and Structures Key Laboratory of Sichuan Province, Southwest University of Science and Technology, Mianyang, China; Federal University of Technology - Parana, BRAZIL

## Abstract

In this paper, eight different contoured cushions (S1-S8) in two categories (flat and wrapped) were designed to study the influence of different contoured cushions on passenger comfort in high-speed trains. Meanwhile, subjective data investigation by the comfort Likert Scale questionnaire and objective physical variables collection by the body-cushion contact pressure test was carried out. In addition, one-way ANOVA and Pearson correlation analysis were performed on the subjective survey and objective test data. The results show that the cushion contours had a significant effect on the subjective evaluation of the overall comfort of the participants, in which the overall comfort below the waist of the separated wrapped cushion S8 has the highest subjective comfort score. The overall comfort of the flat-front bulge type cushion S4 and the local comfort of the thighs and the root of the thighs were rated higher than other flat types. Under the flat cushion, the effect of stature characteristics (mainly weight and hip-width) on the overall comfort subjective ratings was insignificant, and the effect on the contact pressure distribution variables was significant, but the contact pressure distribution variables were not correlated with the comfort ratings. Under the wrapped cushion, the effect of stature characteristics on the overall comfort subjective ratings and contact pressure distribution variables was significant. There were positive and negative correlations between the average peak contact pressure and average contact pressure and comfort ratings, respectively.

## 1. Introduction

**Fig 1:**
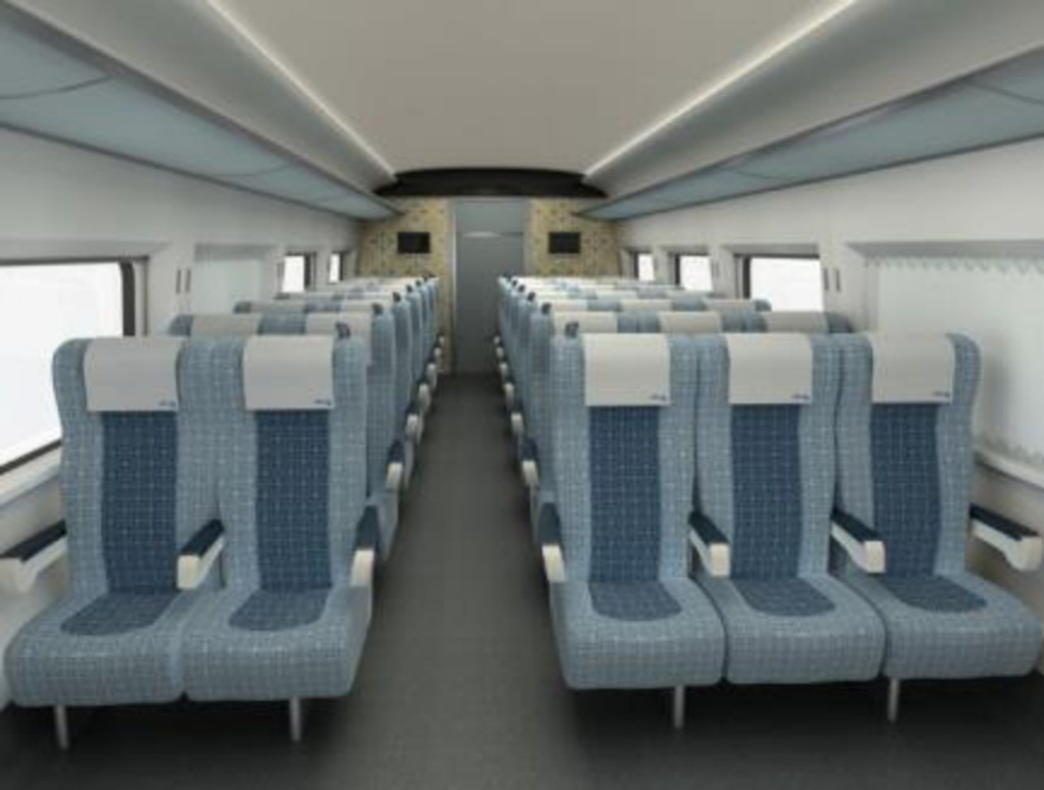
High-speed train (CRH380) interior environment and seats.

By 2020, the operating mileage of high-speed trains in China has reached 35,000 km. High-speed trains have become synonymous with technology, efficiency, safety, and comfort, and they are not only a means of transportation for passengers but also a culture of fast and comfortable travel. People’s demand for high-speed trains has gone beyond functional pragmatism, and more attention has been focused on the comfort and humanistic connotation of the riding environment, such as the CRH380 high-speed train interior environment shown and seats in Fig 1. As the only resting facility that is in contact with passengers’ bodies for a long time, the train passenger seat directly affects the comfort of the ride.

At present, many researchers have carried out studies on the influence of seat shape, material, height, depth, and other factors on ride comfort, involving aircraft [1–3], cars [4, 5], office chairs [6], and wheelchairs [7] and other fields. However, high-speed trains are different from cars, motorcycles, offices, and wheelchairs in terms of riding environment, so the evaluation of seat riding comfort should be different [8]. There are two main methods of seat comfort evaluation, subjective and objective. Zhao et al. [2] investigated the effect of aircraft seat pitch on comfort using a 7-point Likert Scale of comfort combined with seat-body contact surface pressure distribution measurements. However, there is also literature that uses only subjective ratings for comfort analysis, e.g., Vink et al. [9] collected over 10,000 retrospective trip reports to obtain an overall rating of overall comfort (0–10 scale) and subjective feedback on the passenger experience. The subjective comfort evaluation method is only a qualitative evaluation method, which cannot determine which physical factors affect the change in comfort level. Seats are manufactured from a combination of industrial materials with different functional properties and have certain physical properties [10]. Therefore, a quantitative description of the seat-human contact pressure science behavior can help evaluate seat comfort accurately.

The objective evaluation covers several aspects such as sitting behavior, surface electromyography [11], and body pressure distribution. The measurement of pressure distribution at the body-seat contact interface is one of the most common objective methods to analyze or compare the variability of different seats. Kyung and Nussbaum [12] concluded that body-seat interface pressure was closely related to overall comfort ratings by analyzing comfort influencing factors for short-term driving with six-seat combinations for 27 subjects. Wang and Cardoso (2019) [13] investigated seat discomfort by grouping participants by stature, weight, and gender. They indicated that the larger contact area between the human–seat interface led to lower average pressure and peak pressure for the larger BMI participants. Participants with different statures preferred different sizes of seats; relatively, taller participants reported less discomfort in larger seats than in smaller seats, and vice-versa [14]. Akgunduz et al. [15] found that the relationship between contact area, contact force, and peak pressure is also directly related to the anthropometry and the cushion’s ability to deform under the weight of the subject. In fact, the effect of human-chair contact pressure and deformation is closely related to the cushion hardness and material [16, 17], but also to the sitting depth [18] and the footrest height. For aged care seating, armrests were essential for ease of entry and egress [19]. For high-speed trains, the main task of mass travel, to meet the majority of people riding comfort for the purpose, it is difficult to do personalized design. Fiorillo et al. [20] point out that seat-pan design is crucial and could be mainly influenced by two factors: pressure distribution and seat contour. de Mare et al. [7] found that contoured seating bases can provide increased comfort and improved pressure distribution over flat seating bases. The cushion contour had a greater effect on seat pan interface pressure [21]. There is also literature that analyzes the bearing capacity and pressure distribution characteristics of seat cushions through numerical simulation methods [22, 23]. Most of the current studies on the comfort of seating surfaces are based on existing product surfaces or comparative studies of a single contour. Therefore, few studies on the relationship between cushion contours, seat pressure distribution, and passenger comfort/discomfort of high-speed train seats.

Peng et al. [24] performed field trials of the passenger vibration comfort on the Shanghai-Kunming high-speed railway from Changsha to Guiyang, and the results showed that the difference was that the frequency-weighted RMS values measured on high-speed trains were lower than those measured on other vehicles, such as bus, car, tractor, etc. Research on passenger seating comfort has shown that when the vibration reaches the seat is low, comfort is largely determined by static factors [25].

In summary, the cushion is a crucial factor affecting the comfort of high-speed train seats. Therefore, the correlation analysis between cushion contour and comfort was carried out by designing cushions with different contour types and combining subjective and objective methods. Among them, for subjective evaluation, the Likert Scale questionnaire was used to fully collect the overall comfort and local comfort rating data below the waist of the participants. For objective evaluation, the effect of different surface morphology and body shape characteristics on body pressure distribution parameters was tested by a body pressure test system. Based on the one-way ANOVA and Pearson correlation analysis methods, we determined the significant effects of different cushion contours and body types on riding comfort and explored the correlation between the subjective evaluation of comfort and the objective evaluation of pressure distribution parameters. The study can provide a reference for the design of seat cushion surfaces for high-speed trains.

## 2. Methodology

### 2.1 Cushion test condition

**Fig 2:**
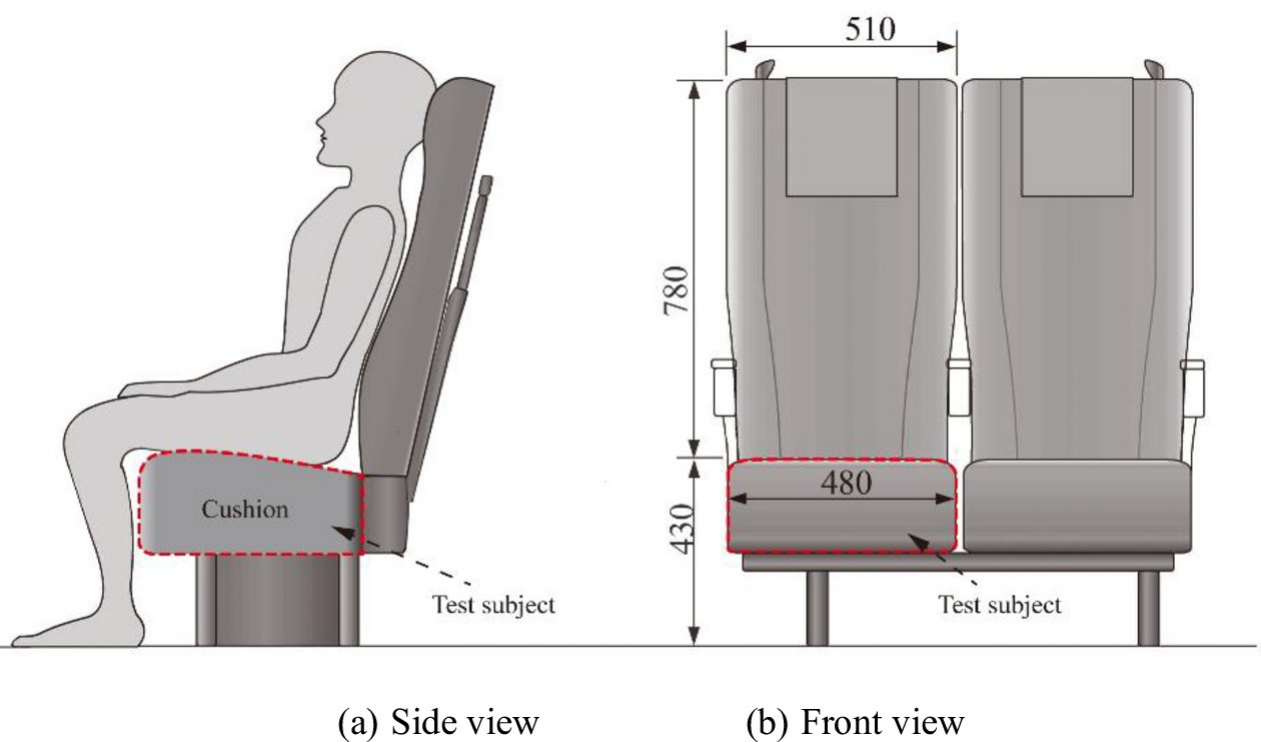
Seat size parameters (unit: Mm). (a) Side view and (b) Front view.

For the seats in the general carriage of high-speed trains, the comfort test experiments of different contoured cushions were carried out in the rail transportation simulation laboratory of Southwest Jiaotong University. To ensure the validity of the test data, the seat dimensions are consistent with the existing high-speed trains as far as possible, except for the different geometry of the seat cushion, and the basic dimensions and sitting posture requirements are shown in Fig 2. Thus, the variable factors are the contour of the cushion, and the fixed factors are the dimensions of the seat, for example, a sitting depth of 450 mm, a sitting width of 480 mm, a sitting height of 430 mm, a backrest height of 780 mm, and a backrest tilt angle of 15 degrees.

### 2.2 Participants

This study comprised 97 human participants. All participants were informed of the aims, the study procedure, and the financial compensation for participation and were asked to follow the rules of the study. The study was approved by the Institution Review Board of Southwest Jiaotong University, and all the procedures followed the regulations for human subject research. Informed consent was obtained from all participants, and the consent was written and recorded in documents. The experimental protocol was divided into program 1 and program 2. Program 1 was a large sample, mainly through the scale to investigate the overall body comfort and local comfort scores below the waist under different cushions. Because of a large amount of pressure distribution data collection, program 1 only conducted a subjective evaluation of the large sample. Program 2, based on program 1, for three different body sizes (weight, hip-width) of the participants, conducted subjective scale data collection and objective physical index collection for the body-cushion contact pressure test, which is listed in Table 1.

**Table 1:** Experimental protocol and sample information.

Program	Evaluation method	Sample numbers	Evaluation indexes and description	
Program 1	Subjective evaluation	Large sample (N = 97)	Overall comfort rating	Whole body
			Local comfort rating	Hip/Ischial tuberosity /Thigh root/Thigh/ Calf /Foot
Program 2	Subjective evaluation	Small sample (N = 6)	Overall comfort rating	Whole body
	Objective evaluation		Average contact area (cm^2^)	Buttock
			Average contact pressure (kPa)	Buttock
			Average peak contact pressure (kPa)	Buttock

#### 2.2.1 Program 1 on participants’ information

**Fig 3:**
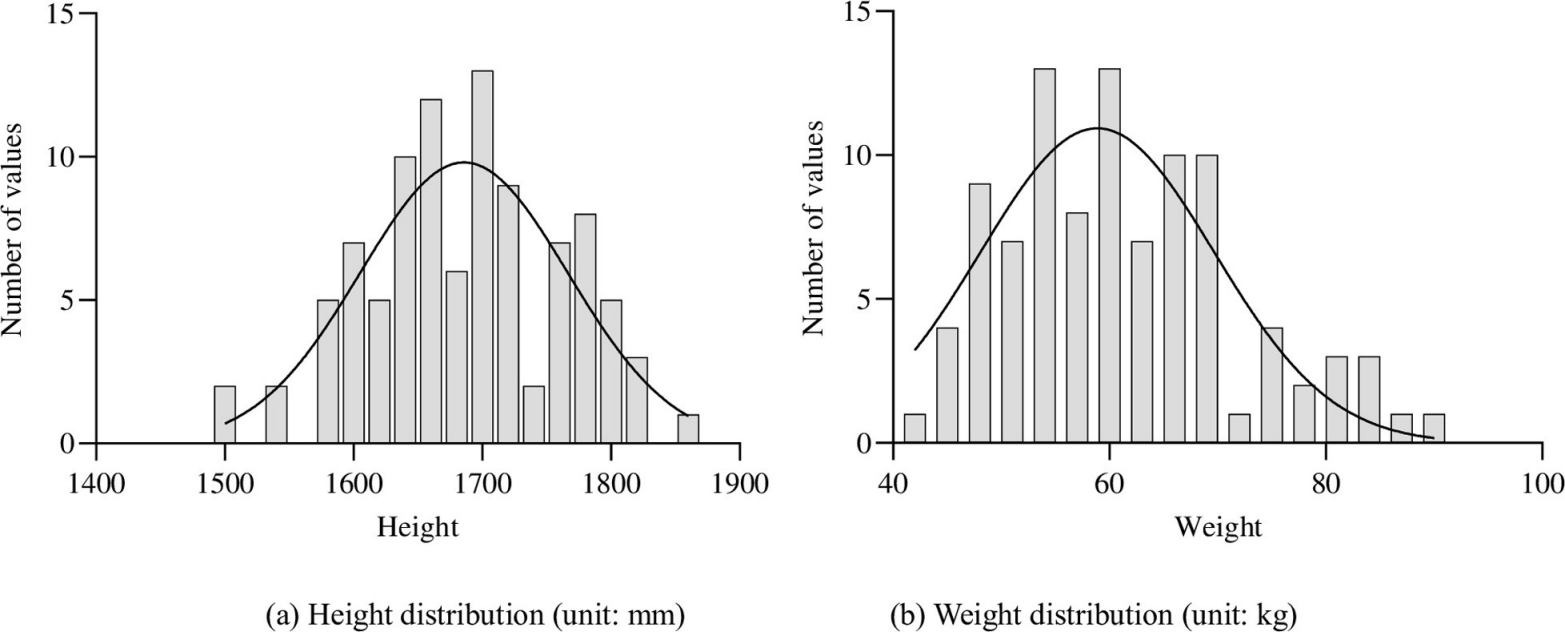
Histogram of subject’s height and weight distribution. (a) Height distribution (unit: mm) and (b) Weight distribution (unit: kg).

To ensure the reliability of the subjective evaluation of cushion comfort, 97 participants were recruited, including 46 males and 51 females, and the participants were mainly university students, graduate students, and teachers. The sample frequencies of height and weight of the participants were statistically analyzed, as shown in Fig 3, respectively. Based on an anthropometric table [26], the heights ranged between the 5th to 98th percentile. In terms of body mass, they ranged between the 20th to 95th percentile. From the curves, it can be seen that the statistical sample basically showed normal distribution characteristics. Thus, the study participants represented a broad range of the population.

#### 2.2.2 Program 2 on participants’ information

It was found in the randomized interviews during the survey in Program 1 that the comfort of different cushions might be influenced by the participants’ weight and sitting hip-width. Therefore, three typical stature types of participants were designed in Program 2, and participants with large, medium and small stature were identified according to the 5th, 50th, and 95th percentiles of the statistical weight sample, respectively, and their basic information is listed in Table 2. The purpose was to test whether there was a relationship between subjective comfort scores and stature characteristics in Program 1 and to explore the correlation between subjective comfort scores and objective parameters of pressure distribution under different stature.

**Table 2:** Basic information of participants in Program 2.

Stature type	Genger	Age	Height/mm	Weight/kg	Hip width/mm
Large stature	Male	29	1790	85	413
	Famle	29	1620	80	400
Medium stature	Male	29	1740	65	375
	Famle	28	1620	62	380
Small stature	Male	28	1720	56	355
	Famle	26	1600	49	342

### 2.3 Cushion geometric contour design

**Fig 4:**
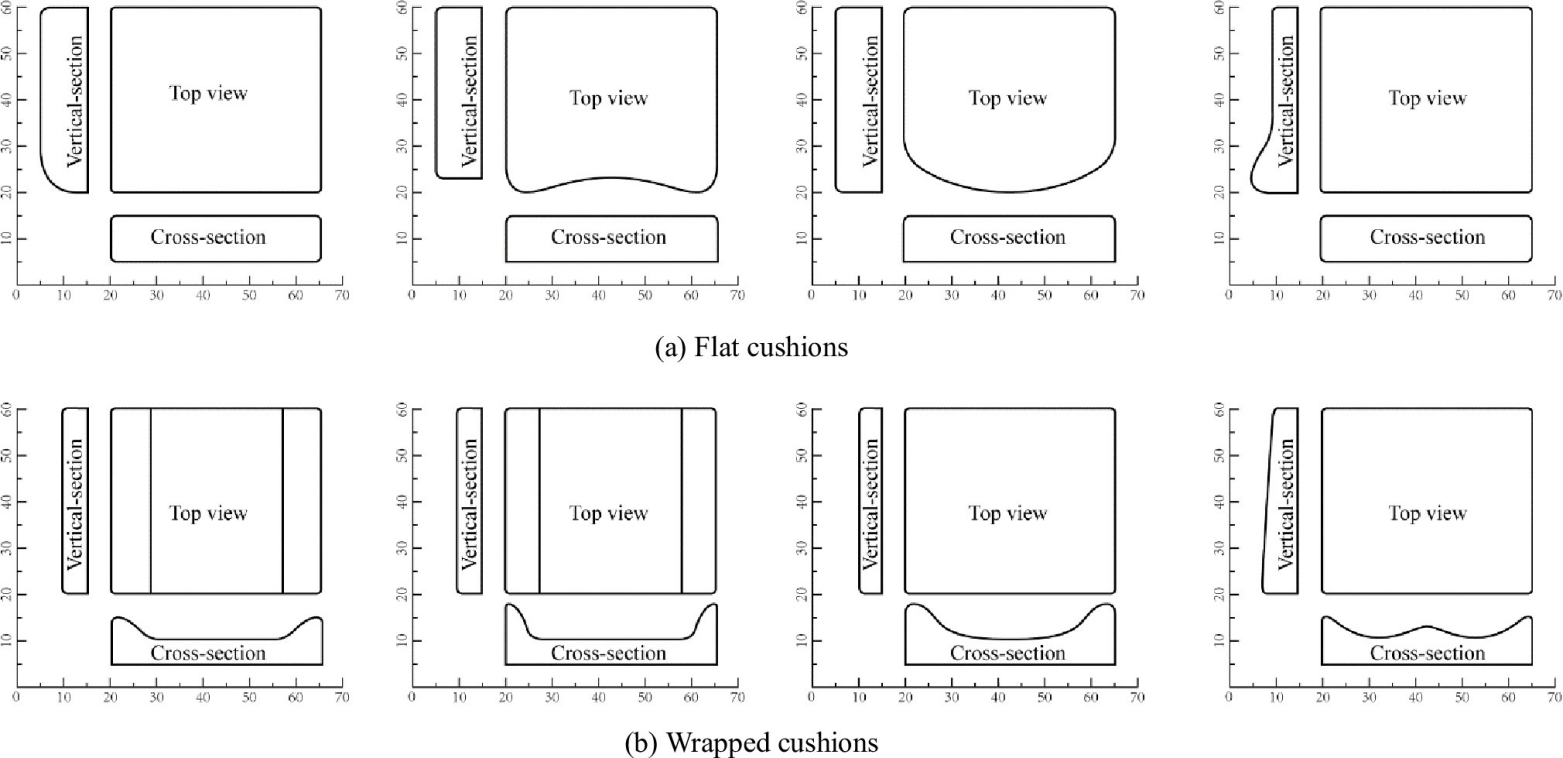
Three views of eight cushion geometry designs (unit: Cm). (a) Flat cushions and (b) Wrapped cushions.

The geometric contour design of the cushion mainly included two types of flat (S1-S4) and wrapped (S5-S8), as shown in Fig 4A and 4B. The experimental participants were asked to experience the cushions S1-S8 in order. The main difference among flat types S1-S4 is the front, S1 is the flat-front down type, S2 is the flat-front concave type, S3 is the flat-front convex type, and S4 is the flat-front drum type. The main difference between wrapped S5-S7 is the degree of wrapping on both sides of the wing, S5 is wrapped-side wing low and slow type, S6 is wrapped-side wing high and steep type, and S7 is wrapped-side wing high and slow type. S8 is a special wrapped type, separated wrapped type.

### 2.4 Cushion material selection and production

**Fig 5:**
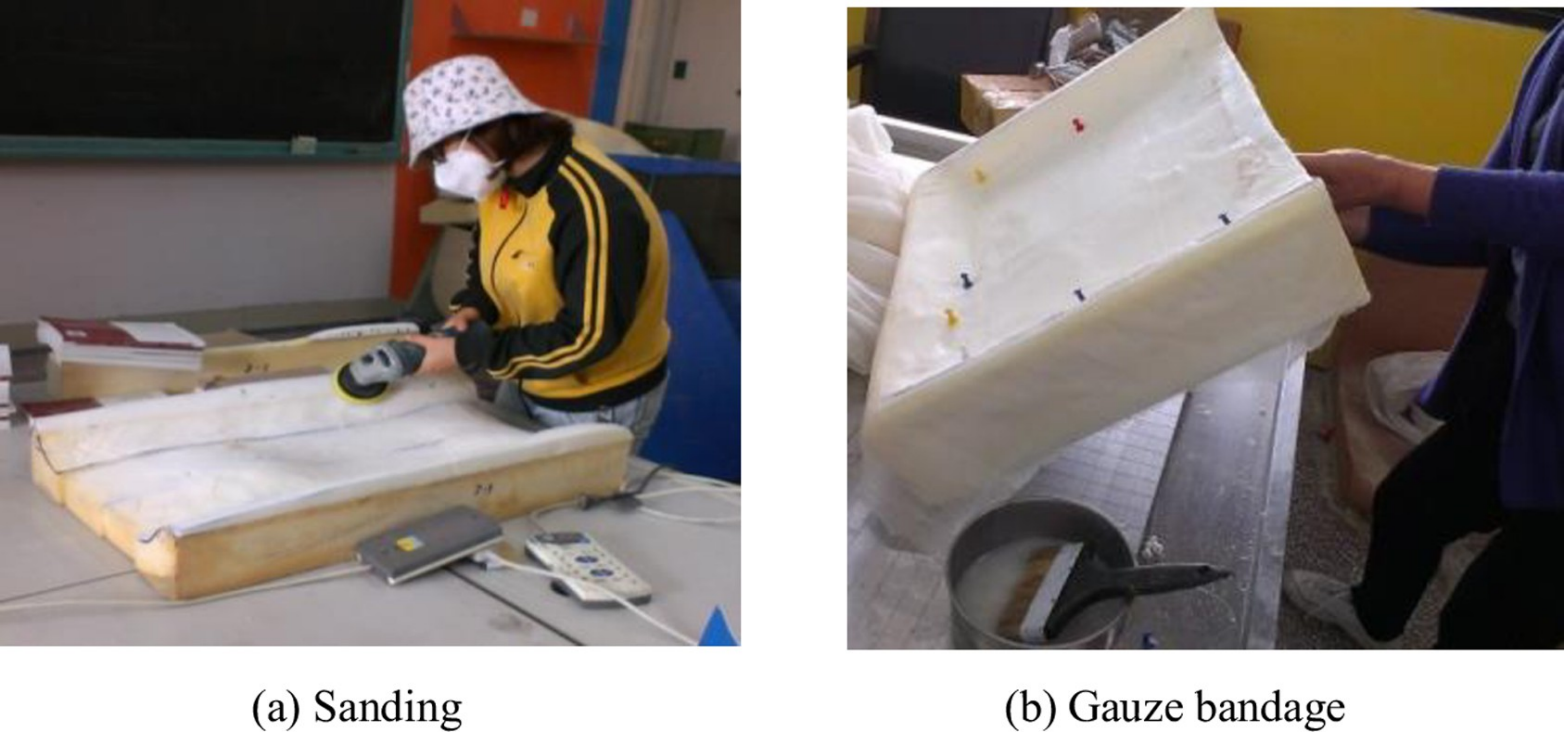
Production process photos. (a) Sanding (b) Gauze bandage.

**Fig 6:**
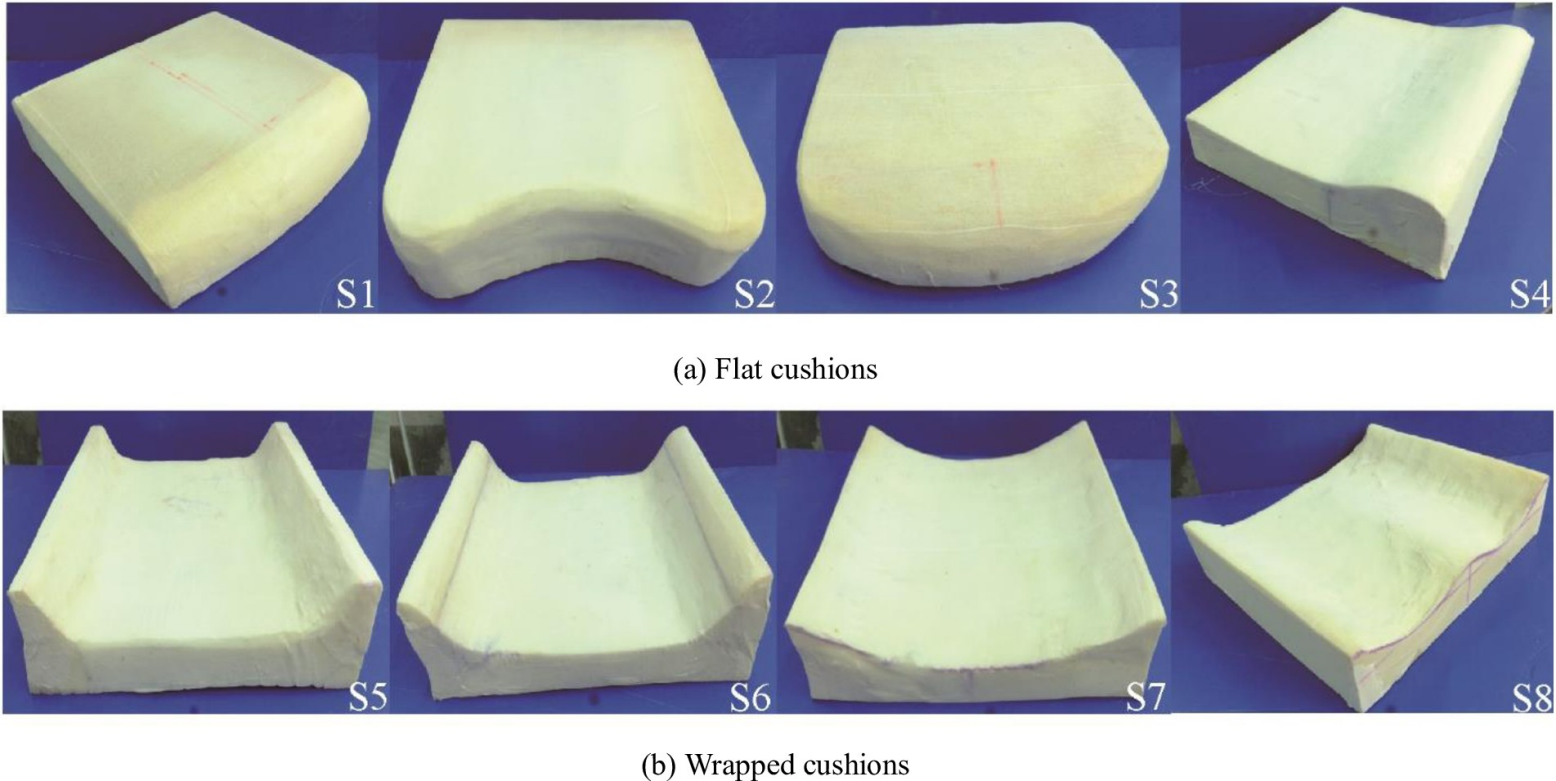
Eight kinds of contoured cushion production molding. (a) Flat cushions and (b) Wrapped cushions.

The material of the cushion is the polyurethane foam used in high-speed train seats. A piece of polyurethane foam size 1.2×0.6m, thickness 0.1m, hardness 45 degrees, can do two cushions. According to the design parameters, the large pieces of polyurethane foam were cut, sanded, trimmed, and tensed with gauze in sequence, as shown in Fig 5. The eight kinds of cushions cut and shaped are shown in Fig 6. The cushion material used in the test is polyurethane foam; each cushion is processed in four steps of measuring, cutting, sanding and masking in turn.

### 2.5 Subjective evaluation method for comfort scale

**Fig 7:**
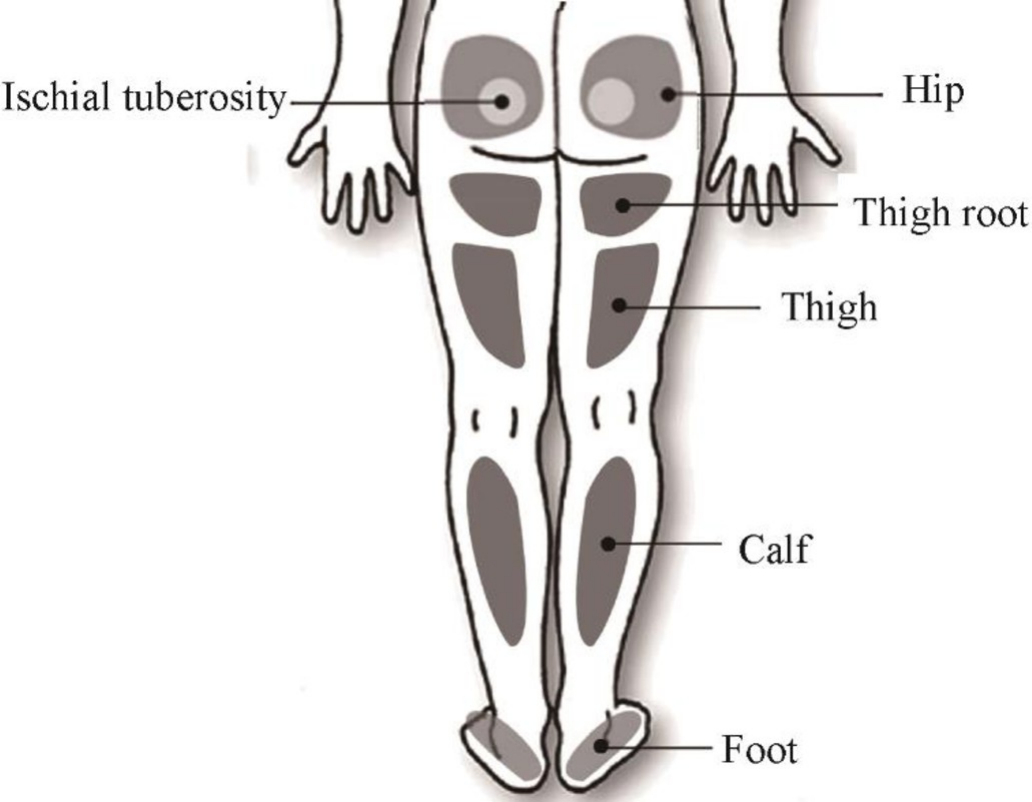
Schematic diagram of the body perception area below the test waist.

**Fig 8:**
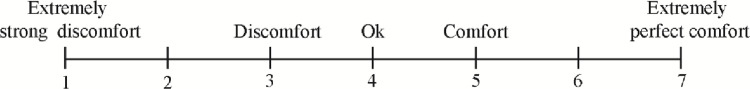
7-point likert scales.

It is difficult for non-specialist participants to understand the contour characteristics of each surface of the seat. The scale design of this experiment is no longer based on the satisfaction evaluation of a feature or parameter of the seat, but is converted into the comfort rating of the key parts of the human body, and the human perception parts are shown in Fig 7. The comfort scale is a 7-point Likert Scale, where the comfort level is from 1 to 7 in descending order, as shown in Fig 8: 1 means "Extremely strong discomfort", 4 means "OK", 7 means "Extremely perfect comfort".

### 2.6 Objective evaluation method for pressure distribution index test

**Fig 9:**
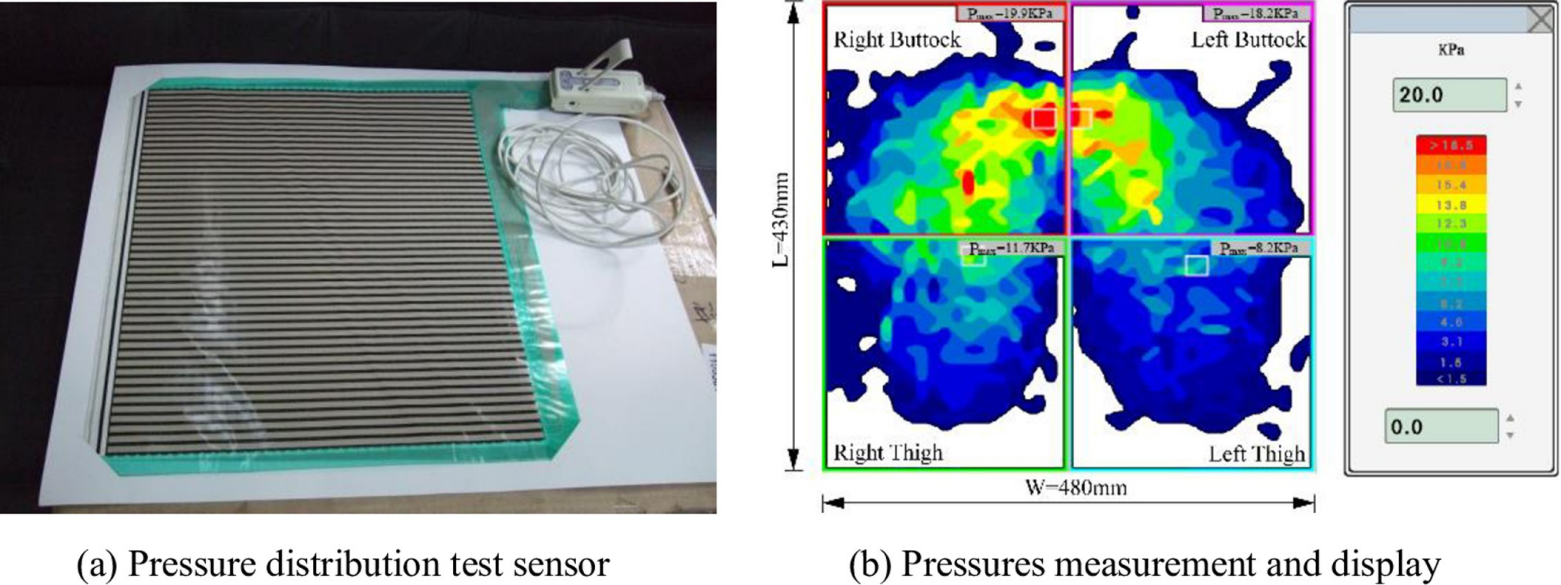
Tekscan production pressure distribution measurement system. (a) Pressure distribution test sensor and (b) Pressures measurement and display.

The human body-cushion contact surface pressure distribution test and the sensor for the United States Tekscan production pressure distribution measurement system are shown in Fig 9. The pressure sensor is a thin film sensor for the seat, with a thickness of 0.35 mm, a single measurement area of 430 mm × 480 mm, and 2064 sensing points. The measurement and analysis system is BPMS Research 7.10, which can realize the main parameters (pressure, area, time) in real-time operation records.

The experiment was conducted by asking participants to be in a state of sensory relaxation while testing the pressure distribution and asking them to maintain a posture on the cushion S1 as shown in Fig 2. After 5 minutes, the pressure distribution recording started while the participants started scoring on the comfort scale. During the test, participants were not able to see the data recordings. After 5 minutes of activity, another test was carried out with cushion S2 followed by cushion S3, and continued until S8. The statistical indicators were average contact area, average contact pressure, and average peak contact pressure.

### 2.7 Data processing methods

One-way ANOVA was conducted using SPASS (version 24) software on the cushion subjective comfort scores and the results of the pressure distribution index test. Among them, the factor in Program 1 was the cushion, with 8 levels, S1-S8, and the observations were the ratings of 97 participants, respectively; the factor in Program 2 was stature, with 3 levels, large stature, medium stature, and small stature, and the observations were the subjective ratings of 6 typical participants and the test data of pressure distribution index, respectively. In the one-way ANOVA, the significance level p was taken as 0.05, and the results were considered significantly different when the calculated p<0.05 was obtained.

## 3. Results

### 3.1 Evaluation under Program 1

#### 3.1.1 Overall comfort rating scale

**Fig 10:**
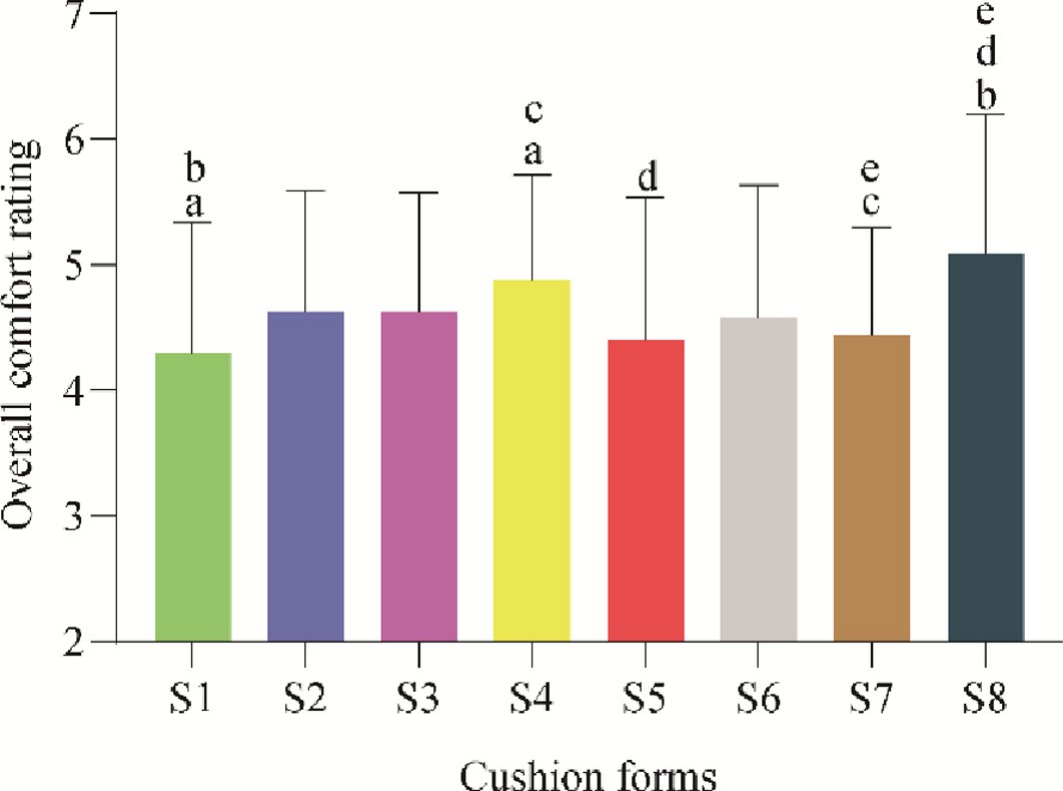
The overall comfort rating score for eight cushion contours averaged over 97 participants, bars with the same letter superscript represent the significant difference (p<0.05).

According to Program 1, 97 participants performed Likert Scale scores for overall comfort after the experience with 8 cushions, and the statistical results are shown in Fig 10. The one-way ANOVA showed that the difference in the effect of different contoured cushions on the overall comfort of the human body was significant (p<0.05). The results of post hoc two-by-two multiple comparisons using the LSD method showed that S4 comfort was significantly higher than S1 and S7, S8 comfort was significantly higher than S1, S5, and S7, and S8 comfort scores had the highest average value.

#### 3.1.2 Local body region comfort rating scale

According to Program 1, 97 participants performed body local comfort scores after experiencing eight cushions, and after one-way ANOVA on the data, the results are listed in Table 3. It can be seen that there are also significant differences (p<0.05) in the reflection of different regions of the body on the comfort of the contoured cushion. For the flat cushion, the design of the outer forward protrusion of the S4 cushion made the comfort of the subject’s thigh root, thigh, and calf significantly higher than other cushions. For the wrapped cushion, results showed that the comfort ratings of the hip, thigh root, thigh, calf, and foot under cushion S8 were significantly higher than those of S5, S6, and S7.

**Table 3:** Local body region comfort rating variables (values in parentheses indicate a standard error).

Local region	Cushion contours								F values (p level)
	Flat cushions				Wrapped cushions				
	S1	S2	S3	S4	S5	S6	S7	S8	
Hip	4.8(0.8)^a^	4.8(0.9)^b^	4.8(0.9)^c^	5(1)^de^	4.8(1)^f^	4.7(1)^dg^	4.6(0.9)^eh^	5.2(1)^abcfgh^	F = 3.0(p<0.05)
Ischial tuberosity	4.8(0.8)^a^	4.8(0.9)^b^	4.8(0.9)	5(1)^cd^	4.8(1)^e^	4.7(0.9)^cf^	4.5(0.9)^abdeg^	5(1)^fg^	F = 2.6(p<0.05)
Thigh root	4.7(0.9)^ab^	4.7(1)^cd^	4.8(0.9)^ef^	5.2(0.9)^acefgh^	4.6(1)^fi^	4.5(1.2g)^j^	4.5(1)^hk^	5.2(1)^bdfijk^	F = 6.6(p<0.05)
Thigh	4.3(1.1)^ab^	4.3(1.1)^cd^	4.6(1.1)^e^	4.8(1)^acfg^	4.3(1.3)^fh^	4.4(1.3)^gi^	4.5(1.1)^j^	5(1.1)^bdehij^	F = 5.0(p<0.05)
Calf	4.3(1.1)^ab^	4.3(1)^cd^	4.4(1)^e^	4.6(1)^acfgh^	4.1(1.1)^efi^	4.2(1.1)^gj^	4.2(1)^hk^	4.6(1)^bdhijk^	F = 3.4(p<0.05)
Foot	4.4(1)	4.6(1)^a^	4.5(1)^b^	4.6(0.9)^cde^	4.2(1.3)^abcf^	4.3(1)^dg^	4.3(1)^eh^	4.6(1)^fgh^	F = 2.7(p<0.05)

Notes: Statistically significant difference (p<0.05) indicated by same letter superscript; NS means no significant difference (p<0.05).

### 3.2 Evaluation under Program 2

#### 3.2.1 Subjective evaluation on S1-S7

**Fig 11:**
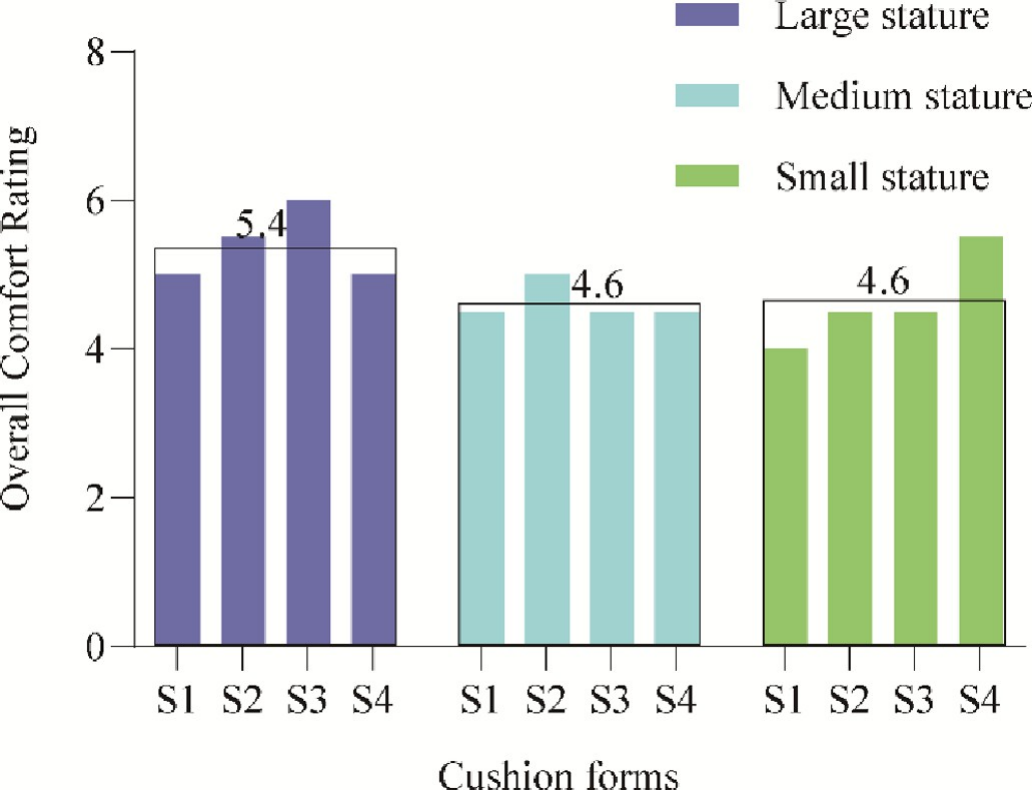
Histogram of average flat cushion comfort scores for three kinds of stature.

*Subjective evaluation on flat cushions(S1-S4)*. The average comfort scores of the flat cushion for the three typical stature are shown in Fig 11. For the flatbed cushion, the average rating of the flat cushion by the participants with large stature was 5.4, which was slightly higher than the 4.6 scores for medium and small stature. The one-way ANOVA results showed no significant difference (p>0.05) in the comfort ratings of the three typical stature participants under the flat cushion, as listed in Table 4.

**Table 4:** Overall body region discomfort rating variables (values in parentheses indicate a standard error).

Cushion contours	Statistic values			F values (p level)
	Large stature	Medium stature	Small stature	
Flat cushions	5.4(0.7)	4.6(0.5)	4.6(0.9)	F = 2.7(p>0.05)
Wrapped cushions	3.5(0.5)^ab^	5.7(0.8)^a^	5.3(0.5)^b^	F = 19.8(p<0.05)

Note: Statistically significant difference (p<0.05) indicated by same letter superscript; NS means no significant difference(p>0.05)

**Fig 12:**
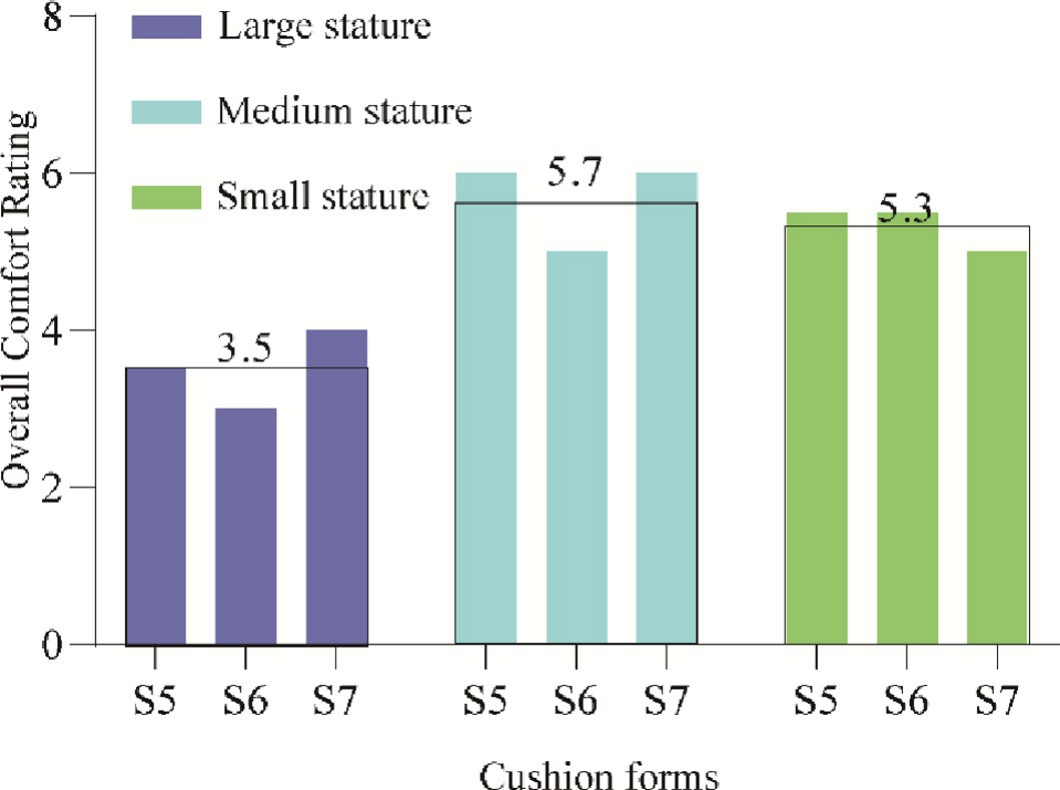
Histogram of average flat cushion comfort scores for three kinds of stature.

*Subjective evaluation on Wrapped cushions(S5-S7)*. The average comfort ratings of the wrapped cushion for the three typical stature of the participants are shown in Fig 12. From the results of the one-way ANOVA, it is clear that for the wrapped cushion, there is a significant difference in the comfort ratings under the three typical stature (p<0.05). Large-statured participants rated the wrapped cushion the lowest, with an average rating of only 3.5, significantly lower than the average ratings of 5.7 and 5.3 for medium and small stature, as listed in Table 3.

The comparison of the subjective ratings of flat and wrapped cushions shows that flat cushions are more suitable for wide and obese large-statured passengers than wrapped cushions; wrapped cushions are more suitable for medium and thin-statured passengers than flat cushions.

#### 3.2.2 Objective evaluation on S1-S7

**Fig 13:**
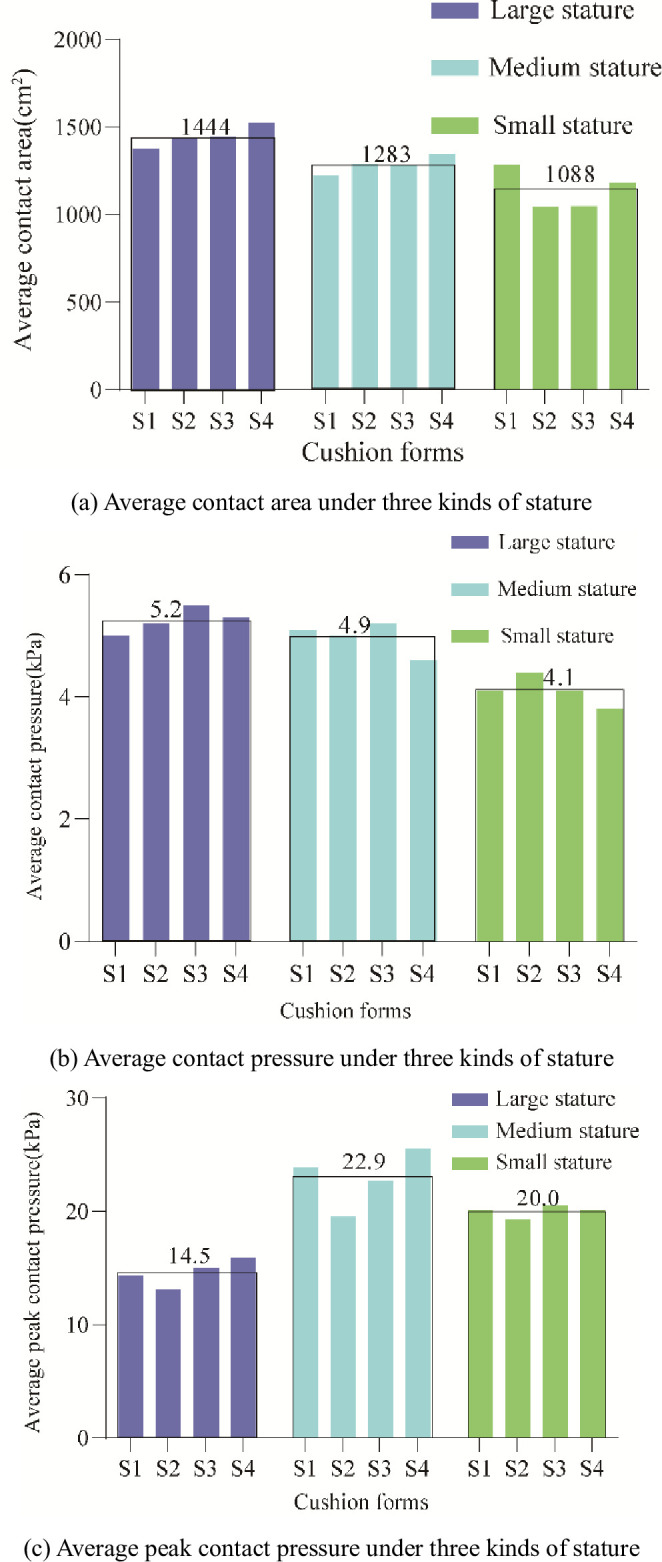
Statistical histogram of pressure distribution parameters of flat cushion under three kinds of stature. (a) Average contact area under three kinds of stature, (b) Average contact pressure under three kinds of stature, and (c) Average peak contact pressure under three kinds of stature.

**Fig 14:**
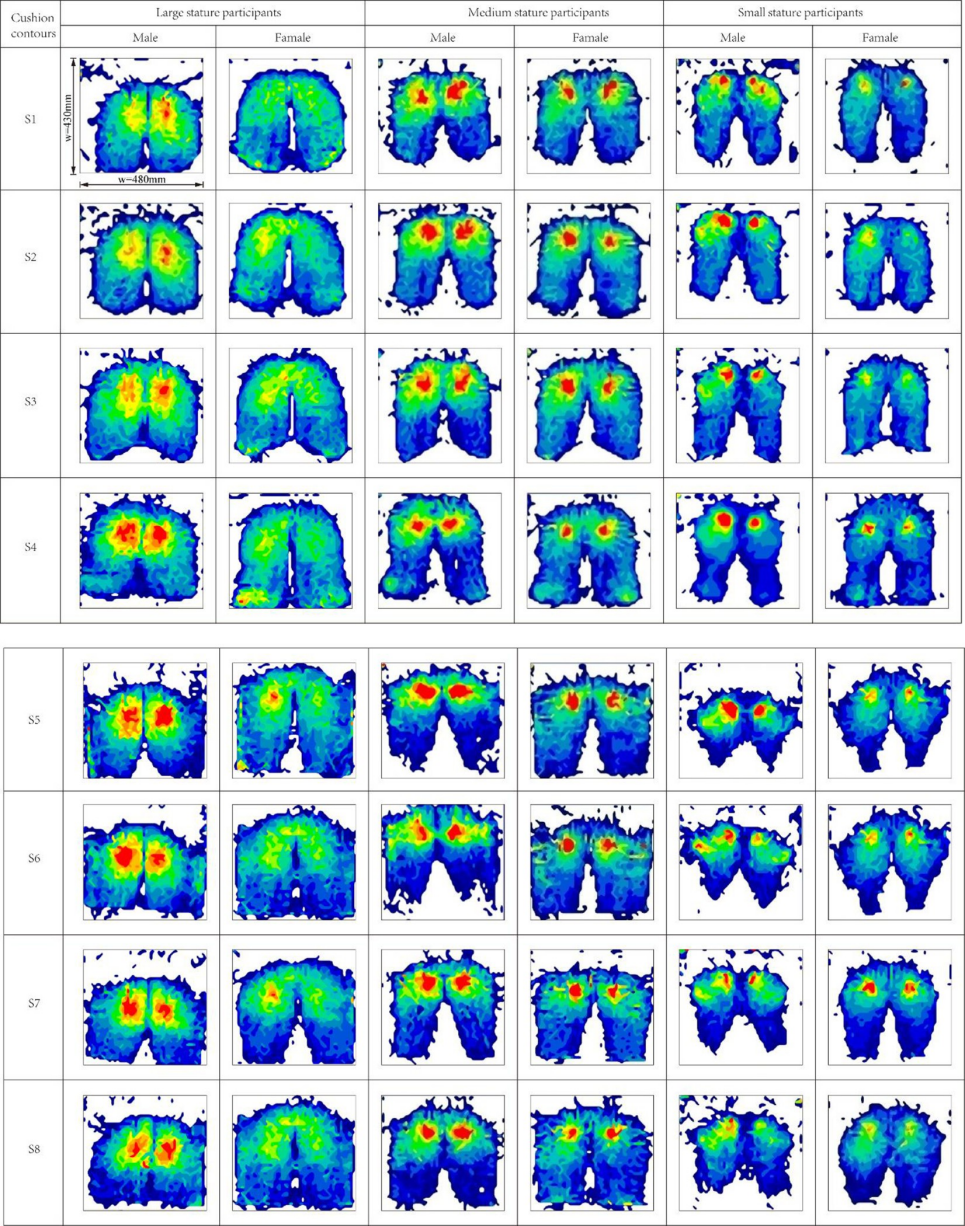
Body-cushion contact pressure distribution cloud map.

*Objective evaluation on flat cushions(S1-S4)*. The average statistics of the three indexes of average contact area, average contact pressure, and average peak contact pressure were measured by the Tekscan pressure distribution measurement system for participants of different stature, as shown in Fig 13A–13C. The results of the one-way ANOVA are listed in Table 5. It can be seen that there is a significant difference (p<0.05) between stature characteristics on all three indexes. In Fig 13A, the average contact area and average contact pressure of participants with large stature are significantly higher than those with small stature. In contrast, the average peak contact pressure is significantly lower than that of small and medium stature. The reason is that participants of large stature with large bodyweight and wide hips have plump gluteus maximus as a cushion, which helps to disperse the pressure at the sciatic node, resulting in a large average contact area and high average pressure but low average peak pressure, as shown in Fig 14. The participants of small stature were lighter, so the average pressure was lower than that of the medium-statured participants, but the average peak contact pressure was not much different from that of the medium-statured participants.

**Table 5:** Objective experimental test values at three stature under flat cushions (values in parentheses indicate a standard error).

Objective indexes	Test values			F values (p level)
	Large stature	Medium stature	Small stature	
Average contact area	1444(63.1)^ab^	1283(61.0)^ac^	1088(73.2)^bc^	F = 34.2(p<0.05)
Average contact pressure	5.2(0.3)^a^	4.9(0.3)^b^	4.1(0.3)^ab^	F = 32.7(p<0.05)
Average peak contact pressure	14.5(2.5)^ab^	22.9(2.9)^a^	20.0(5.0)^b^	F = 3.8(p<0.05)

Note: Statistically significant difference (p<0.05) indicated by same letter superscript; NS means no significant difference(p>0.05)

**Fig 15:**
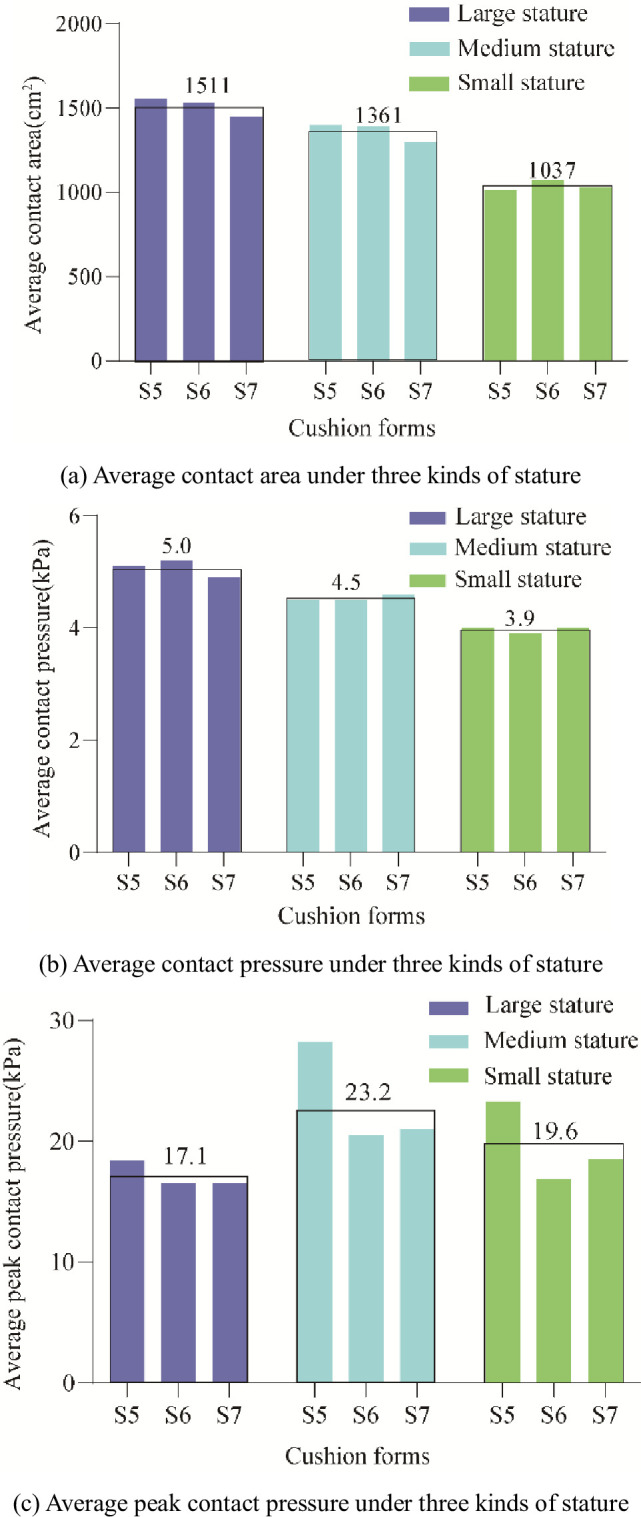
Statistical histogram of pressure distribution parameters of wrapped cushion under three kinds of stature. (a) Average contact area under three kinds of stature, (b) Average contact pressure under three kinds of stature, and (c) Average peak contact pressure under three kinds of stature.

Each figure in Fig 15 has the same size of 430 mm × 480 mm, as shown in Figure (S1, Male), in which the pressure of different strengths is indicated by different colors, with light blue indicating pressures less than 1.5 kPa and red indicating pressures greater than 20 kPa. The dimensions of the images were also uniformly labeled to reflect the test results of large/medium/small statures. In addition, the text in the figures after software processing was also processed to ensure readability. Test pressure data can be found in the Supporting Information.

*Objective evaluation on Wrapped cushions(S5-S7)*. For the wrapped cushion, the average statistics of the three indexes of average contact area, average contact pressure, and average peak contact pressure were measured for participants of different stature, as shown in Fig 15A–15C. The results of the one-way ANOVA are listed in Table 6. The average contact area and average contact pressure of large participants were significantly higher than those of medium and small participants in Fig 15A and 15B. In contrast, the average peak contact pressure was significantly lower than that of medium participants but not significantly different from that of small participants because large stature participants had large body weight but also large hip width, which facilitated pressure dispersion. At the same time, small participants had small hip widths but were also lightweight. Therefore, the measured peak pressures were not significantly different from those of the large participants, as shown in Figs 15C and 14.

**Table 6:** Objective experimental test values at three stature under wrapped cushions (values in parentheses indicate a standard error).

Objective indexes	Test values			F values (p level)
	Large stature	Medium stature	Small stature	
Average contact area	1511(108.7)^ab^	1361(87.0)^ac^	1037(107.3)^bc^	F = 34.2(P<0.05)
Average contact pressure	5.0(0.3)^ab^	4.5(0.1)^ac^	3.9(0.2)^bc^	F = 32.7(P<0.05)
Average peak contact pressure	17.1(2.7)^a^	23.2(4.0)^a^	19.6(4.6)	F = 3.8(P<0.05)

Note: Statistically significant difference (p<0.05) indicated by same letter superscript; NS means no significant difference(p>0.05).

*Objective evaluation comparison analysis on flat and wrapped cushions*. Average contact area comparison. The average contact areas of large and medium stature under the flat cushion were 1444 cm^2^ and 1283 cm^2^, which were smaller than the average contact areas of 1511 cm^2^ and 1361 cm^2^ under the wrapped cushion, while the average contact areas of small stature were not much different, 1088 cm^2^ and 1037 cm^2^, as shown in Fig 12. It can be seen that for medium and large stature participants, the wrapped cushion played a wrapping effect, while it did not play a wrapping effect for small stature participants due to their thin stature.

Average contact pressure comparison. The average contact pressures of 5.2 kPa, 4.9 kPa, and 4.1 kPa for large, medium, and small stature under flat cushion are higher than the average contact pressures of 5.0 kPa, 4.5 kPa, and 3.9 kPa for large, medium, and small stature under the wrapped cushion, due to the increase in average contact area resulting in lower average contact pressure.

Average peak contact pressure comparison. The average peak contact pressure of 14.5 kPa for the large stature under the flat cushion was significantly lower than the average peak contact pressure of 17.1 kPa for the large stature under the wrapped cushion, and the peak contact pressure of the small and medium stature did not change much, indicating that the wrapped cushion produced a significant squeezing sensation for the large stature participants.

#### 3.2.3 Subjective and objective evaluation on S8

Although the cushion S8 belongs to the wrapped type, due to the central separation design, and S5-S7 cushion is different. Cushion S8 under even the large stature passenger sitting position, the two inner thigh parts are not easy to squeeze each other, with the effect of separation wrapped. Therefore. Cushion S8 has higher overall comfort ratings of 6.0, 7.0, and 5.5 for large, medium, and small stature, respectively, and its average value is also higher than that of S5-S7 (see Fig 13), which is also consistent with the results in Fig 10. For large, medium, and small stature participants, the average contact area and average contact pressure also decreased gradually due to the sequential decrease in hip width and weight. However, for the average peak contact pressure, both medium and small stature was greater than the average peak pressure under large stature participants, as listed in Table 7.

**Table 7:** Subjective and objective evaluation index value on S8.

Evaluation method	index	Stature types		
		Large stature	Medium stature	Small stature
Objective evaluation	Average contact area(cm^2^)	1547	1435	1116
	Average contact pressure(kPa)	4.8	4.2	3.7
	Average peak contact pressure(kPa)	15.9	23.4	21.9
Subjective evaluation	Overall comfort rating	6.0	7.0	5.5

### 3.3 Correlations among ratings of subjective and objective

Pearson correlation coefficients of the subjective ratings and pressure indexes were carried out for two types of cushions, the flat type (S1-S4) and the wrapped type (except cushion S8 because of its specificity), and the results are listed in Table 8. From the table, it can be seen that under the flat cushion, the average peak contact pressure and the average contact pressure have no correlation with the comfort rating, but for the wrapped cushion, the average peak contact pressure and the comfort rating exist in a positive correlation, that is, the average peak contact pressure, the better the comfort; the average contact pressure and the comfort rating have a negative correlation, the higher the average contact pressure, the lower the comfort rating.

**Table 8:** Pearson correlation coefficients of the subjective ratings and pressure variables.

	Overall comfort rating score	Average peak contact pressure	Average contact pressure
Overall comfort rating score	1\1		
Average peak contact pressure	-0.234\0.543*	1\1	
Average contact pressure	0.272\-0.550*	-0.147\-0.027	1\1

Note:* p<0.05(2-tailed). The data on the left side of "\" is the flat cushion contour, and the data on the right side of "\" is the wrapped cushion contour.

## 4. Discussion

Recent literature shows that interface contact pressure is useful for investigating discomfort during seating [21, 27–29]. This study provides comprehensive insights into the effects of different cushion contours on the contact pressure distribution at the human body-cushion interface, and the discomfort levels in the whole body and local body regions.

Program 1 surveyed overall and local body comfort scales, and from the one-way ANOVA, it was found that the cushion S8 has the highest subjective comfort score, followed by the cushion S4, and the cushion S1 has the lowest score. Among them, the difference between S1 and S4 is in the front-end design of the seat cushion, which is the front-end downward and upper drum types, respectively, and the comfort score of S4 is significantly higher than that of S1 (p<0.05). However, the S1 is the more popular cushion type in office seating [6]. For office conditions, the front of the seat is a desk, and the front is tilted downward to maintain an upright or forward-leaning working posture [30]. Train and plane passengers usually lean on the seat backrest [31–33], so in the test, the participants were asked to keep their backs leaning on the backrest with a 15° inclination, and the front-bulge type S4 could have a more stable focus on the hips. This prevents the body from slipping, reducing the support of the legs and giving a comfortable feeling. This also shows that the comfort evaluation of the seat is closely related to the riding environment [8, 13]. Wang et al. [13] found that changing the inclination angle and height of the backrest also has an impact on the body pressure distribution on the cushion, which in turn affects the comfort score; that is, sitting posture has a significant impact on comfort [21, 34]. Therefore, the riding environment and sitting posture should be consistent when evaluating ride comfort. The aircraft seat comfort evaluation focuses on the problem of the front and rear seat pitch [2, 31], which is different from high-speed trains, where the flow of passengers in the train is more mundane, with the front and rear seats facing each other, so the train seats themselves are more important than the pitch. The separation design in the middle of the cushion S8 is slightly tilted upwards with the front, so that the participants, even those with larger statures, do not easily squeeze each other on both inner thighs, and the two inner thighs will be more comfortable, which is consistent with the evaluation of the same contour wheelchair [7], so the best comfort score. However, for the wrapped cushion S5-S7 in the questionnaire, follow-up found that different participants feel different, the overall participants tend to have a little wrapped cushion, the degree of wrapping too much will produce a sense of inner thigh squeeze, making people feel uncomfortable, the degree of wrapping S5-S7 is actually related to the figure (hip-width and weight).

The purpose of the design of program 2 is to test whether the comfort score in program 1 is related to stature, on the other hand, and to examine whether there is a direct link between the objective index and the subjective score of the two types of cushions under different statures. Participants with different statures preferred different sizes of seats; relatively, taller participants reported less discomfort in larger seats than in smaller seats, and vice-versa [3, 14]. Cardoso [13] and Wang et al. [14] investigated seat discomfort by grouping participants by stature, weight, and gender. They indicated that the larger contact area between the human–seat interface led to lower average pressure and peak pressure for the larger BMI participants. Usually, for tall people, sitting in a standard height seat, the feet will be in full contact with the ground, supporting the load on the lower legs and part of the thighs, resulting in pressure on the soles of the feet, stress on the knee joints, and increased pressure at the sciatic nerve nodes, producing a sense of discomfort. For people of short stature, when the seat height is too high and they cannot fully step on the ground, they cannot reach the ground with both feet flat, which will create a feeling of uneasiness [3]. For the flat cushion, S1-S4 scores were averaged. Based on the one-way ANOVA, it is clear that the relationship with stature is not obvious because neither large nor small stature has the feeling of wrapping on both sides. For the wrapped cushion, the large stature obviously felt the sense of squeezing (except S8), which is more suitable for the participants with small and medium stature. Therefore, the high-speed train may set up a wide-wrapped cushion in the VIP business compartment.

Based on the correlation analyses of the human body-cushion contact pressure distribution, the pressure variables were correlated with overall region discomfort ratings [21]. Romano et al. [35] argued that the lower the peak pressure at the ischial tuberosity, the higher the comfort rating of the cushion, however, this does not take into account the impact of the subject’s stature. Under the flatbed cushion, the average peak contact pressure and average contact pressure did not correlate with the comfort score because the average contact pressure was correlated with stature (weight) [34], and the average contact pressure decreased sequentially from large stature to small stature, as shown in Figs 13B and 15B, while the peak contact pressure was correlated with the thigh muscle at the ischial tuberosity, and the peak pressure at the ischial tuberosity was not concentrated for the stature with obese hips, as shown in Fig 14. Therefore, the peak pressure of large stature is lower than that of small and medium stature. Akgunduz et al. [15] found that there is a strong correlation between the perceived comfort level and the measured average peak contact pressure and average contact pressure. However, there is no significant difference between the subjective comfort of flat cushion and stature characteristics (see Table 4), so there is no correlation between objective variables and subjective scales. There is a positive correlation between the average peak contact pressure and comfort score for the wrapped cushion and a negative correlation between the average contact pressure and comfort score. This conclusion is inconsistent with the finding of Li et al. [36]. The reason is that this paper takes into account the differences in body stature and cushion contour. Due to the small average peak contact pressure of large stature, the wrapped cushion makes the inner thighs feel strongly squeezed, so the comfort score is low. The medium stature peak pressure is caused by the superimposed effect of body weight and thigh muscle at the hip ischial tuberosity, and there is a comfortable wrapped feeling, so it will get the highest comfort score. Wang et al. [15] found that the preferred pressure proportion on the seat pan depended on the sitter’s body size. Therefore, the design layout of high-speed train seats according to VIP, first class, and second class seats is reasonable to meet the comfort needs of passengers of different statures.

## 5. Conclusion

One-way ANOVA and Pearson’s correlation analysis of the survey and measurement data were conducted for eight different types of high-speed train passenger cushions with different surface contours, taking into account the body form characteristics of the participants, and using a combination of subjective evaluation by the comfort Likert Scale questionnaire and objective evaluation by the body-cushion contact pressure test, to draw the following conclusions:

The cushion contours had a significant effect on the subjective evaluation of the overall comfort of the participants. Among them, the cushion S8 has the highest subjective comfort score, followed by the cushion S4, and the cushion S1 has the lowest score. The separated wrapped cushion S8 increased the contact area and lowered the average contact pressure, which could improve the comfort of the hip, thigh, thigh root, calf, and foot. The front-bulge type S4 could improve the comfort of the thigh and thigh root. However, the comfort ratings of the other cushions were all stature dependent, i.e., for large stature participants, flat cushions (S1~S3) were superior to wrapped cushions (S5~S7), but the results were reversed for medium and small stature.

Under the flat cushion, the effect of stature characteristics (mainly weight and hip-width) on overall comfort subjective scores was not significant, and the effect on body pressure distribution parameters was significant, with the average contact pressure of small stature significantly lower than that of large and medium stature participants, and the average peak contact pressure of large stature participants, significantly lower than that of medium and small stature participants; under the wrapped cushion, the effect of stature characteristics on overall comfort subjective scores and body pressure distribution parameters were significant, with the average contact pressure of large, medium and small stature participants significantly lower in turn, and the average peak contact pressure of large stature participants significantly lower than that of medium stature participants.

Pearson correlation analysis showed that the average peak contact pressure and average contact pressure did not correlate with the comfort score for flat cushions, but for wrapped cushions, the average peak contact pressure and average contact pressure correlated significantly with the comfort score. There was a positive correlation between the average peak contact pressure and comfort score; there was a negative correlation between the average contact pressure and comfort score.

In future research work, the effect of the superposition effect of long-term riding and micro-vibration of high-speed trains on passenger body fatigue will be considered, and the effect of seat variables such as seat backrest and footrest on riding comfort will also be increased, to improve the seat design parameters for comfortable riding on high-speed trains.

## Supporting information

S1 File Table Objective experimental test dates at three statues. (DOCX)
