# Supplementary material for: Influences of cushion contour on passenger comfort and interface pressure in high-speed train
Source: PLoS One. 2023 Feb 13;18(2):e0276900. doi: 10.1371/journal.pone.0276900 (PMC9925231; doi:10.1371/journal.pone.0276900)
Supplement: S1 File — (DOCX) [file pone.0276900.s001.docx]

Table Objective experimental test dates at three statues

| Cushions | Statures | Participants | Peak contact pressure*/*kPa | Average contact pressure*/*kPa | Contact area/cm*^2^* |
| --- | --- | --- | --- | --- | --- |
| S1 | LSP | P1 | 16 | 4.9 | 1338 |
|  |  | P2 | 12.5 | 5.1 | 1414 |
|  | LMP | P3 | 24.5 | 5.2 | 1208 |
|  |  | P4 | 23.3 | 4.9 | 1235 |
|  | SSP | P5 | 23.1 | 4.4 | 1123 |
|  |  | P6 | 17.1 | 3.8 | 1041 |
| S2 | LSP | P1 | 14.3 | 5.3 | 1449 |
|  |  | P2 | 11.8 | 5 | 1424 |
|  | LMP | P3 | 19.9 | 5.2 | 1245 |
|  |  | P4 | 19 | 4.7 | 1332 |
|  | SSP | P5 | 23.9 | 4.6 | 1040 |
|  |  | P6 | 14.6 | 4.1 | 1044 |
| S3 | LSP | P1 | 17.5 | 5.8 | 1484 |
|  |  | P2 | 12.5 | 5.1 | 1404 |
|  | LMP | P3 | 22.1 | 5.2 | 1231 |
|  |  | P4 | 23.3 | 5.1 | 1330 |
|  | SSP | P5 | 27.9 | 4.1 | 1087 |
|  |  | P6 | 13 | 4 | 1008 |
| S4 | LSP | P1 | 18.3 | 5.4 | 1516 |
|  |  | P2 | 13.4 | 5.2 | 1527 |
|  | LMP | P3 | 28.6 | 4.7 | 1316 |
|  |  | P4 | 22.3 | 4.5 | 1374 |
|  | SSP | P5 | 20.4 | 3.9 | 1124 |
|  |  | P6 | 19.7 | 3.7 | 1237 |
| S5 | LSP | P1 | 19.7 | 5.1 | 1483 |
|  |  | P2 | 17.1 | 5 | 1628 |
|  | LMP | P3 | 27.8 | 4.6 | 1317 |
|  |  | P4 | 28.6 | 4.4 | 1479 |
|  | SSP | P5 | 28.6 | 4.2 | 890 |
|  |  | P6 | 18 | 3.7 | 1129 |
| S6 | LSP | P1 | 19.6 | 5.5 | 1422 |
|  |  | P2 | 13.3 | 4.8 | 1633 |
|  | LMP | P3 | 18.7 | 4.5 | 1329 |
|  |  | P4 | 22.2 | 4.4 | 1452 |
|  | SSP | P5 | 16.5 | 4 | 986 |
|  |  | P6 | 17.3 | 3.8 | 1156 |
| S7 | LSP | P1 | 18.6 | 5.2 | 1365 |
|  |  | P2 | 14.3 | 4.5 | 1532 |
|  | LMP | P3 | 20.6 | 4.7 | 1250 |
|  |  | P4 | 21.4 | 4.5 | 1343 |
|  | SSP | P5 | 17.1 | 4 | 956 |
|  |  | P6 | 19.9 | 3.9 | 1102 |
| S8 | LSP | P1 | 19.6 | 5 | 1429 |
|  |  | P2 | 12.2 | 4.5 | 1665 |
|  | LMP | P3 | 22.1 | 4.2 | 1362 |
|  |  | P4 | 24.6 | 4.2 | 1508 |
|  | SSP | P5 | 26.6 | 3.7 | 1073 |
|  |  | P6 | 17.1 | 3.7 | 1159 |

Note: LSP, LMP, and SSP represent large, medium, and small stature participants, respectively
